# Practical Uranalysis and Urinary Diagnosis

**Published:** 1895-06

**Authors:** 


*Practical Uranalysis and Urinary Diagnosis.* By CHARLES W. PURDY, M.D. Pp. xiv., 357. Philadelphia: THE F. A. DAVIS CO. London: F. J. REBMAN. 1894.

Dr. Purdy's book is deserving of high praise. He describes a method devised by himself of rapidly determining the amounts after precipitation of the phosphates, chlorides, and sulphates present by means of the centrifugal machine. In testing for albumen by heat, we prefer to boil the top layer of the column of fluid, and not the whole of the fluid in the tube. He regards the acetic acid and ferrocyanide of potassium test as the next best one for general use. The convenience of picric acid as a precipitant for all classes of proteids, and an aid therefore in differentiating peptones, deserves more appreciation. The salicyl-sulphonic acid test for albumen will, we believe, be found a most delicate and trustworthy one, and useful in the differentiation of albumoses and peptones; no doubt it will obtain a more extended notice in future editions. The author has modified Fehling's solution in order to render it more stable.

This volume is well illustrated, most of the plates being borrowed from Peyer. There is a short but well-written account of the clinical features of the most important diseases of the kidney, and the book ends with a capital chapter on the examination of the urine for life assurance. The work is a highly practical one, evidently written by a man of large experience. The binding, printing, and illustrations are admirable.

